# Non-coding RNA in rhabdomyosarcoma progression and metastasis

**DOI:** 10.3389/fonc.2022.971174

**Published:** 2022-08-11

**Authors:** Farah Ramadan, Raya Saab, Nader Hussein, Philippe Clézardin, Pascale A. Cohen, Sandra E. Ghayad

**Affiliations:** ^1^ Department of Biology, Faculty of Science II, Lebanese University, Beirut, Lebanon; ^2^ Université Claude Bernard Lyon 1, Lyon, France; ^3^ INSERM, Unit 1033, LYOS, Lyon, France; ^4^ Department of Chemistry and Biochemistry, Laboratory of Cancer Biology and Molecular Immunology, Faculty of Science I, Lebanese University, Hadat, Lebanon; ^5^ Department of Anatomy, Cell Biology and Physiology, Faculty of Medicine, American University of Beirut, Beirut, Lebanon; ^6^ Department of Pediatric and Adolescent Medicine, American University of Beirut Medical Center, Beirut, Lebanon; ^7^ Aix-Marseille University, INSERM 1263, INRAE 1260, C2VN, Marseille, France

**Keywords:** rhabdomyosarcoma, non-coding RNAs, miRNA, lncRNA, circRNA, rRNA, prognosis, therapeutic targets

## Abstract

Rhabdomyosarcoma (RMS) is a soft tissue sarcoma of skeletal muscle differentiation, with a predominant occurrence in children and adolescents. One of the major challenges facing treatment success is the presence of metastatic disease at the time of diagnosis, commonly associated with the more aggressive fusion-positive subtype. Non-coding RNA (ncRNA) can regulate gene transcription and translation, and their dysregulation has been associated with cancer development and progression. MicroRNA (miRNA) are short non-coding nucleic acid sequences involved in the regulation of gene expression that act by targeting messenger RNA (mRNA), and their aberrant expression has been associated with both RMS initiation and progression. Other ncRNA including long non-coding RNA (lncRNA), circular RNA (circRNA) and ribosomal RNA (rRNA) have also been associated with RMS revealing important mechanistic roles in RMS biology, but these studies are still limited and require further investigation. In this review, we discuss the established roles of ncRNA in RMS differentiation, growth and progression, highlighting their potential use in RMS prognosis, as therapeutic agents or as targets of treatment.

## Introduction

Rhabdomyosarcoma (RMS) is a pediatric cancer currently classified into four histological subtypes with embryonal (ERMS) and alveolar (ARMS) being the most common. While ERMS represents 60% of RMS cases, it is less clinically aggressive than ARMS (1, 2). The latter is often characterized by the presence of a fusion oncoprotein, namely paired box 3-forkhead Box O1 (PAX3-FOXO1) or PAX7-FOXO1 that exhibit a more potent transcriptional activation of PAX3/7 targets and are expressed at higher levels compared to wild-type PAX3 and PAX7 (2–4). Presence of these fusion oncoproteins, and more particularly PAX3-FOXO1, is also associated with disease aggressiveness, tumor invasion and metastasis (5–8). Since the absence of a fusion oncoprotein in certain ARMS cases makes them indistinguishable from ERMS in terms of prognosis and overall survival, a better classification of RMS tumors is based on fusion status rather than histology, dividing it into fusion-positive (FP-)RMS or fusion-negative (FN-) RMS (2, 9).

While FP-RMS is associated with chromosomal translocations that encode altered transcription factors, FN-RMS is notably associated with loss-of-heterozygosity (LOH) in the chromosomal region 11p15.5 which harbors tumor suppressive *IGF2*, *H19*, and *CDKN1C* genes, chromosomal gains and losses, *TP53* mutations, and increased expression of the *HRAS* oncogene (10, 11). Concerning the latter, mutations in the proto-oncogenes encoding RAS family members are one of the most commonly occurring mutations in FN-RMS where findings reveal that gain-of-function mutations in *HRAS* are a common molecular event that occurs during ERMS development (12). Furthermore, alterations in *NRAS*, *KRAS* and *HRAS* genes were mostly found in ERMS compared to fusion-negative ARMS tumors whereas FP-RMS tumors lacked these mutations (13). Despite histological and molecular differences between FP-RMS and FN-RMS, both subtypes exhibit defective or incomplete differentiation (14). FP- and FN-RMS cells consistently express myogenic markers, but they fail to complete differentiation into mature skeletal muscle cells due to mechanisms that are starting to be elucidated, such as an impaired transactivation function of MyoD, a master transcription factor associated with muscle cell differentiation (15).

**Figure 1:**
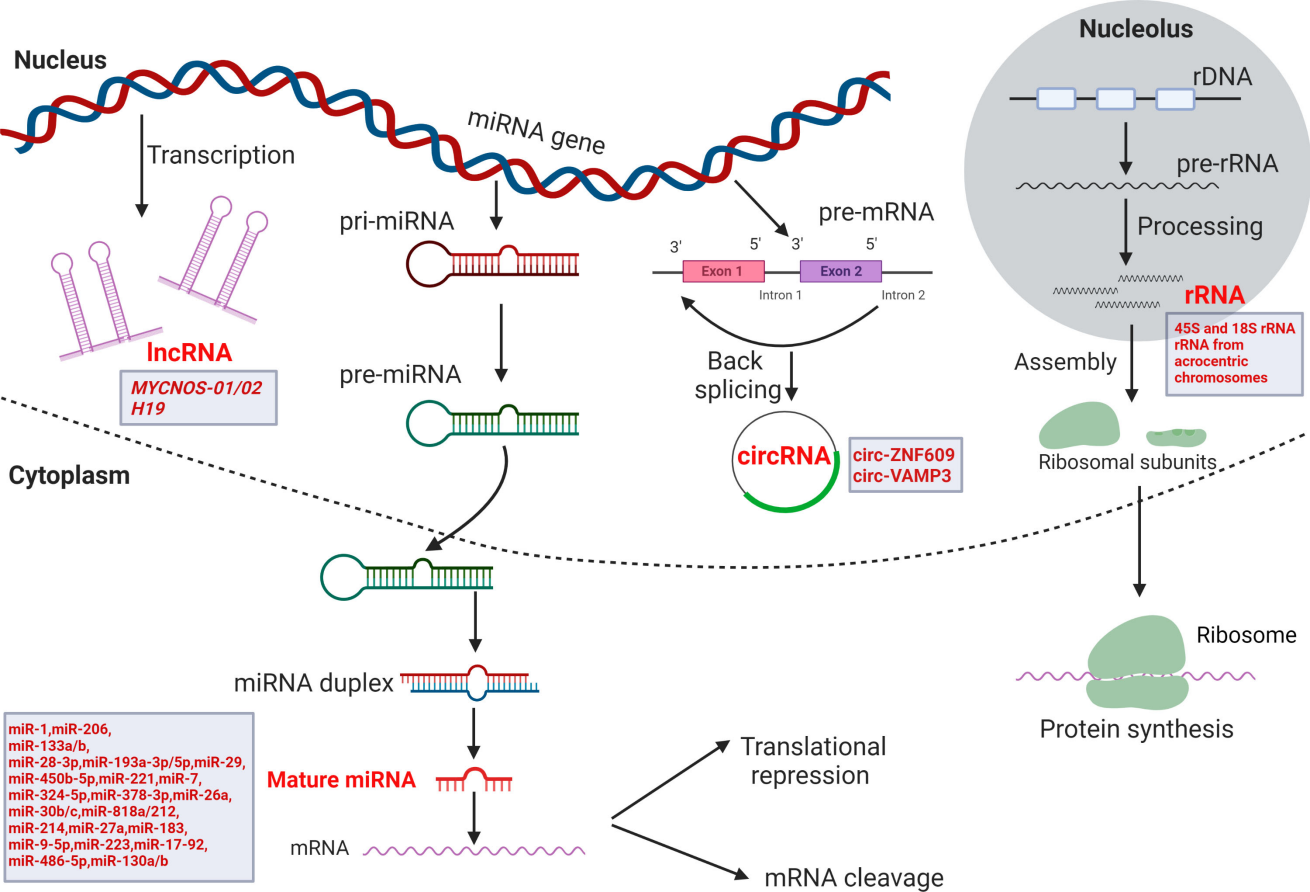
The different types of ncRNA that are implicated in RMS progression and metastasis. From left to right: lncRNA can be transcribed from different regions of DNA including enhancers and promotors; miRNA are transcribed first as pri-miRNA sequences which are subsequently processed into pre-miRNA and transported into the cytoplasm to complete maturation and bind to target mRNA for silencing or degradation; a pre-mRNA can be back-spliced to form a circRNA; within the nucleolus, rDNA can encode rRNA that regulate protein synthesis within the ribosomal complex. The ncRNA implicated to date in RMS biology and progression are represented within boxes. Created BioRender.com.

Moreover, dysregulated expression of non-coding RNA (ncRNA) including microRNA (miRNA), long non-coding RNA (lncRNA), circular RNA (circRNA) and ribosomal RNA (rRNA), is associated with incomplete myogenic differentiation and enhanced proliferation of RMS cells (16–19). **Figure 1** represents a brief and general overview of the different classes of ncRNA that are implicated to date in RMS biology, and their biogenesis within the cell.

MiRNA are short ncRNA, with an average length of 22 nucleotides, which regulate cellular pathways and processes, including those involved in cancer growth and progression. The term oncomiR applies to a miRNA that inhibits the expression of tumor suppressive genes and further promotes cell growth and survival; thus, tumor-suppressive miRNA are those whose expression in cancer cells and the tumor microenvironment is often down-regulated to escape anti-cancer mechanisms (20, 21). Several miRNA have dual roles in cancer depending on the type of tumor (22). MiRNA biogenesis begins in the nucleus where a gene is first encoded into a primary transcript (pri-miRNA) which is processed by Drosha, an RNAse III enzyme, associated with the RNA-binding protein Pasha into a precursor pre-miRNA (**Figure 1**). Within the cytoplasm, the pre-miRNA is further processed by the Dicer enzyme into a functionally active mature miRNA (23) (**Figure 1**). MiRNA can downregulate target expression levels based on the degree of complementarity to the target mRNA, mostly between the seed region of the miRNA and the 3’ untranslated region (3’UTR) of the target mRNA, which can subsequently result in target degradation (24, 25). On the other hand, when only partial complementarity exists, the miRNA can repress the translation of mRNA, reducing only the protein levels of their target (26, 27). Some miRNA are expressed in a tissue-specific manner indicating a role in cellular differentiation and an association with certain diseases (28). A miRNA can act individually in targeting gene expression and producing important effects, but a concordance in the upregulation or downregulation of different miRNA under specific conditions reveals that multiple miRNA can regulate levels of several transcripts in a cooperative manner leading to augmented outcomes (29–31). In cancer, many miRNA are either enriched or downregulated with the majority of miRNA genes showing decreased expression compared to normal tissue suggesting that most miRNA act by inhibiting tumorigenesis (32, 33). In fact, rapidly emerging evidence suggests that miRNA loss-of-function contributes to cancer growth and progression highlighting the potential of miRNA replacement therapies as treatment strategies (34).

While miRNA modulate the expression of their target mRNA, lncRNA act by regulating gene expression at both the transcriptional and post-transcriptional levels, and some alter post-translational protein function by modulating protein phosphorylation, acetylation or glycosylation (35, 36). They are larger than 200 nucleotides and modulate a myriad of functions in many cellular processes (36). Furthermore, many lncRNA have been shown to interact with chromatin-modifying complexes and can organize chromatin structure into active or inactive domains; this is one way by which nuclear-enriched lncRNA regulate myogenesis (37). In fact, certain lncRNA can regulate the binding of transcription factors to myogenic loci including MyoD (38). They play significant roles in either promoting or suppressing tumor growth and may therefore also act as potential biomarkers with prognostic significance (39). LncRNA may also indirectly regulate mRNA expression by regulating the activity of other ncRNA, they may compete with miRNA in mRNA binding and interestingly, emerging evidence shows that they may also act as miRNA precursors (40, 41).

Also associated with various aspects of skeletal muscle development are circRNA. For instance, circLMO7, which was found to have the most downregulated expression in adult muscle relative to embryonic muscle tissue based on a circRNA expression analysis, is able to sustain myoblast survival and inhibit their differentiation (42). CircRNA are eukaryotic transcripts that result from non-canonical back-splicing causing exon circularization and a very stable structure with no 5′ and 3′ free termini (**Figure 1**). Their function remains largely undefined but have been shown to bind to miRNA, possibly modulating their function (43). Studies have focused on their role in regulating cell cycle progression and contributing to cancer cell proliferation (43).

While the aforementioned are small ncRNA, rRNA are much larger in size and are essential for ribosome function, protein synthesis and overall cell survival (44). They are mostly transcribed in the nucleoli and along with proteins constitute the ribosomal subunits that control protein synthesis (**Figure 1**). While ribosome biogenesis is considered a house-keeping process beginning in the nucleolus, studies have shown that it can be modulated in a cell type-specific manner. Cancer cells, for example, can alter rRNA synthesis rate as they demand higher ribosome activity and increased protein synthesis (45). In skeletal muscle, rRNA and ribosomes can regulate gene expression and contribute to myogenesis by promoting the biogenesis of myogenic markers. In one study, DEAD-Box RNA (DDX27) helicase, which is required for skeletal muscle growth, was demonstrated to regulate myogenesis by regulating rRNA maturation (46).

Here we review the identified roles of different ncRNA that may be associated with RMS tumor progression and the development of metastasis and those that aid in better prognosis of RMS. We also highlight the potential use of those ncRNA in therapeutic strategies against RMS including pro-differentiation therapies.

## MiRNA implicated in RMS

MiRNA can regulate RMS progression and may serve as clinical biomarkers. They can act by directly regulating myogenic-regulatory factors thereby affecting muscle differentiation (47). **Table 1** summarizes the different miRNA described below and that are implicated in RMS differentiation, tumor progression and prognosis.

**Table 1:** miRNA players in rhabdomyosarcoma.

miRNA	Role in RMS	Expression levels in RMS (compared to NSM)	Validated targets in RMS (direct or indirect)	Effect on RMS biology	Ref.
miR-1*	Tumor suppressive	Downregulated	PAX3/7, c-Met, CCND2, ZNF281, HDAC4, SMARCD1	Promotes differentiation Cell cycle arrest Migration inhibition Inhibition of proliferation	(31, 48–50)
miR-206*	Tumor suppressive	Downregulated	PAX3/7, NOTCH3, CCND2, c-Met, IL-4, HDAC4, Smad3	Promotes differentiation Inhibition of proliferation Migration inhibition	(31, 48, 49, 51–59)
miR-133a/b*	Tumor suppressive	Downregulated	HDAC4, SMARCD1	Inhibition of RMS growth	(60)
miR-28-3p	Tumor suppressive	Downregulated	EZRIN	Inhibition of RMS migration and invasion Decrease in cell adhesion to endothelial cells Downregulation of RMS proliferation Cell cycle arrest Promotion of differentiation	(61, 62)
miR-193a-3p	Tumor suppressive	Downregulated	N/A	Inhibition of RMS migration Decrease in cell adhesion to endothelial cells	(61, 62)
miR-193a-5p	Tumor suppressive	Downregulated	N/A	Inhibition of RMS migration and invasion Downregulation of RMS proliferation Cell cycle arrest Promotion of differentiation	(61, 62)
miR-29 family	Tumor suppressive	Downregulated	GEFT, YY1, CCND2, E2F7, HDAC4, Smad3	Inhibition of proliferation, migration and invasion Inhibition of RMS growth Promotes myogenic differentiation	(31, 57, 63–65)
miR-450b-5p	Tumor suppressive	Downregulated	ENOX2, PAX9	Inhibition of RMS growth Promotes myoblast differentiation	(66)
miR-221	Tumor suppressive	Downregulated	CCND2, CDK6, ERBB3	Induces apoptosis Inhibits migration and invasion	(67)
miR-7	Tumor suppressive	Downregulated	ITGA9	Inhibition of cell proliferation Reduction of tumor growth and metastasis *in vivo* Inhibition of invasion *in vitro*	(68)
miR-324-5p	Tumor suppressive	Downregulated	ITGA9	Inhibition of cell proliferation Reduction of tumor growth and metastasis *in vivo*	(68)
miR-378-3p	Tumor suppressive	Downregulated	IGF1R	Inhibition of RMS migration Induction of apoptosis Cell cycle arrest Enhancement of differentiation	(69)
miR-26a ~	Tumor suppressive	Downregulated	N/A	Correlation with progressive disease	(59)
miR-30b/c ~	Tumor suppressive	Downregulated	N/A	N/A	(59)
Myogenic cocktail miR-818a/212	Tumor suppressive	N/A	N/A	Pro-myogenic effects Increase myotube fusion index Inhibition of migration Inhibition of proliferation Reduction of tumor size *in vivo*	(70)
miR-214	Tumor suppressive	Downregulated	N-ras	Inhibition of tumor growth Promotes differentiation Induction of apoptosis Inhibition of colony formation Inhibition of xenograft growth	(71)
miR-27a	Tumor suppressive/ OncomiR	Upregulated	PAX3-FOXO1, RARA, RXRA	Promotes RMS cell proliferation	(72, 73)
miR-183	OncomiR	Upregulated	EGR1, PTEN	N/A	(74)
miR-9-5p	OncomiR	Upregulated	N/A	Correlates with poor outcome, enhanced migration and metastasis	(75, 76)
miR-223	OncomiR	Upregulated	N/A	Associates with increased epithelial-mesenchymal transition and inflammatory pathways	(77)
miR-17-92 cluster	OncomiR	Upregulated	N/A	N/A	(78)
miR-486-5p*	OncomiR	Upregulated	N/A	Promotes RMS proliferation, invasion and colony formation	(67, 79)
miR-130a/b	OncomiR	Upregulated	PPARG	Promotes proliferation	(80)

*refers to myomiR; N/A refers to undetermined targets in RMS; ~ serum miRNA; NSM, normal skeletal muscle.

## Tumor suppressive MiRNA

### Muscle-specific miRNA or myo-miRNA implicated in RMS

Muscle-specific miRNA, expressed in both cardiac and skeletal muscle, referred to as myo-miRNA or myomiR, regulate normal skeletal muscle differentiation by promoting myogenesis (47). They seem to be essential in muscle differentiation as evidenced by the fact that their dysregulated expression inhibits proper skeletal muscle growth and offers diagnostic potential in skeletal muscle dystrophies (81, 82). Their expression is regulated by myogenic transcription factors essential for skeletal muscle differentiation including MYOD1, MYOG, MYF5, MYF6 and MEF2 (83). Canonical myomiR currently include the miR-1 family (84). This latter can be divided into two groups based on the seed region: miR-1/miR-206 and miR-133a/b (81). MyomiR miR-1 and miR-206 differ by 3 nucleotides outside the seed region and miR-206 is the only known myomiR that is specific to skeletal muscle tissue (51, 85). Later members of the myomiR family include miR-208a, miR-208b, miR-499 and miR-486 (86). miR-486 is considered as “muscle-enriched” since its expression is not limited to muscle tissue, but it is nonetheless considered by some studies as a myomiR (83). miR-208a (which is cardiac specific) and miR-499 serum levels can be associated with myocardial damage in cardiovascular disease (87). The role of miR-1, miR-206 and miR-133 in skeletal muscle differentiation and RMS biology is described below.

### MiR-1 and miR-206

**Figure 2:**
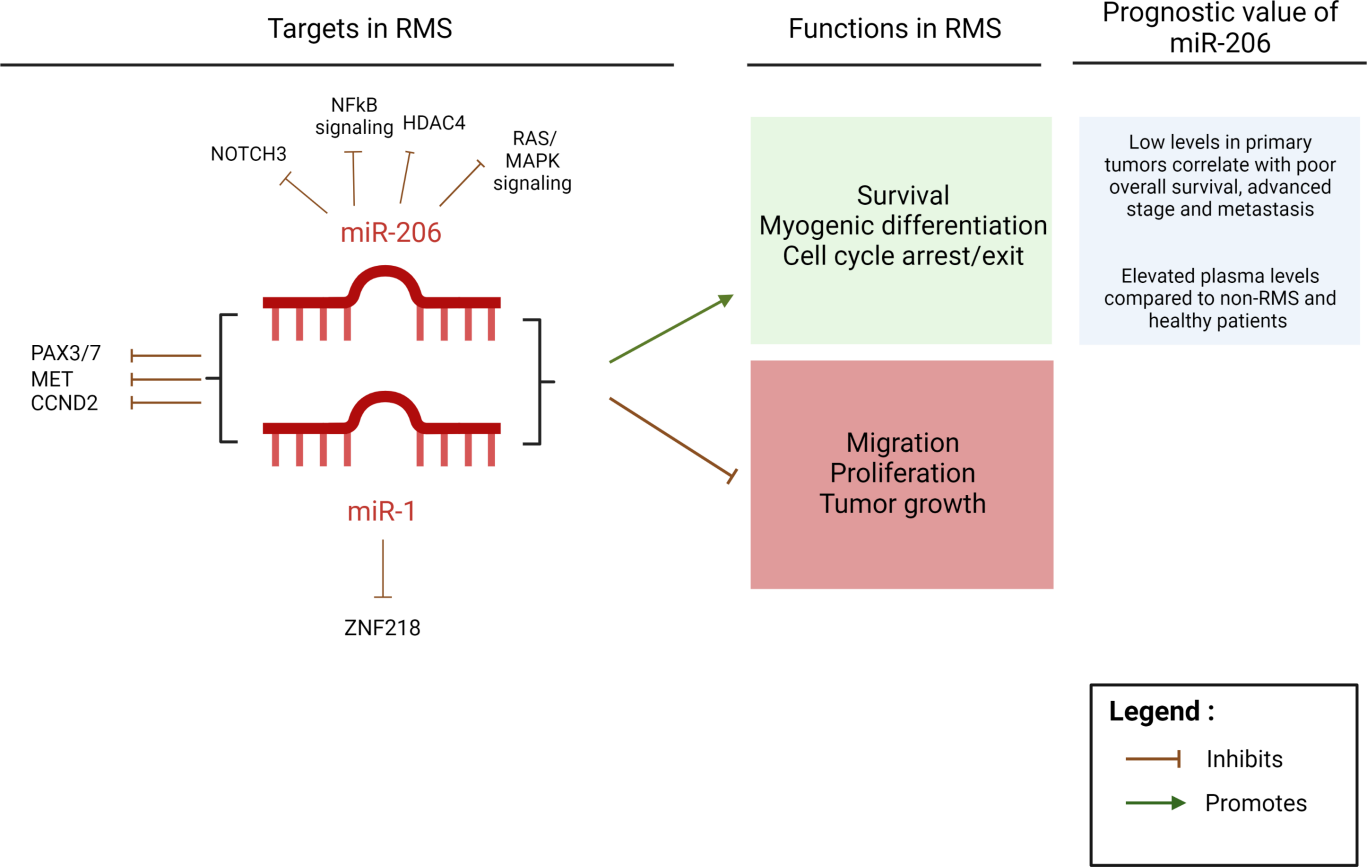
miR-1 and miR-206 targets and functions in RMS. Schematic diagram of miR-1 and miR-206 common and unique targets that have been demonstrated in RMS, and their functions and the prognostic value of miR-206 in RMS. Created with BioRender.com.

While the upregulated expression of myomiR, particularly miR-1 and miR-206, in RMS cell lines relative to other sarcoma subtypes supports the myogenic origin of RMS, their downregulated expression in RMS tumors compared to normal skeletal muscle tissue suggests that they may be important tumor-suppressive agents in RMS (31, 47) (**Figure 2**). In fact, both miR-1 and miR-206 levels are downregulated in primary ARMS and ERMS tumors relative to normal skeletal muscle (47). This may be secondary to the downregulation in myogenic factors, such as MyoD which normally activates miR-1 and miR-206 expression, but is itself inactivated in RMS (88). Of note, in comparison studies, normal skeletal muscle would not be the ideal control for RMS as the former displays complete myogenic differentiation; a better control would therefore be skeletal myoblasts or satellite cells. In turn, miR-1 levels are higher in FP-RMS compared to FN-RMS which may be due to elevated myogenic factor levels in FP-RMS (52). Additionally, the ectopic upregulation of miR-1 mediates cell cycle arrest in FN-ERMS only, associated with the upregulation of myogenin (MyoG) (60). As for miR-206 expression in the two fusion subtypes, analysis of a large number of primary RMS samples revealed that low miR-206 levels correlate with poor overall survival, advanced stage and metastatic disease upon diagnosis in FN-RMS and expressing it in both FN-RMS and FP-RMS proved to have therapeutic potential (52). On the other hand, while miR-206 levels were downregulated in RMS tissues, plasma levels of circulating miR-206 were found to be upregulated in RMS patients, namely ARMS, compared to both healthy donors and non-RMS patients with other tumor types suggesting its potential use as prognostic biomarker in serum samples of RMS patients (53, 89). While in this study localization of serum miR-206 is not specified, this could be a mechanism of cellular release and disposal of tumor suppressive miRNA which can be mediated *via* extracellular vesicles, including exosomes. In fact, tumor cells may maintain their oncogenic properties by exosomal release of tumor suppressive miRNA that becomes elevated in patient sera (90).

In addition to myogenic factors, myo-miR expression can be regulated by SNAIL proteins. For example, MiR-206 can promote myogenic differentiation in ARMS cells upon silencing of *SNAIL* family zinc finger 1 (54). SNAIL is a transcription factor with established roles in regulating RMS growth and differentiation (54, 91). SNAIL expression levels are higher in ARMS tumors compared to ERMS, which is associated with worse prognosis and its levels correlate with those of PAX3/7-FOXO1 (92). One of the mechanisms of action of SNAIL is regulating the expression of miRNA. In one study, silencing *SNAIL* was able to induce myogenic differentiation in the FP-RMS cell line Rh30 through the upregulation of myogenic factors which then upregulate miR-206 (54). The regulation of myogenic differentiation was found to be owed to miR-206 since transfection of Rh30 cells with the *miR-206* precursor supported this differentiation whereas miR-206 inhibition reversed the effect induced by *SNAIL* knockdown (54).

MiR-1/miR-206 expression may also be regulated by PAX transcription factors. This is demonstrated by the fact that in FP-RMS Rh30 cell line, subclones lacking the expression of transcription factor PAX7 displayed decreased proliferation and migration accompanied with enhanced expression of several myomiR including miR-1-3p, miR-133a-3p, miR-133b, and miR-206 compared to PAX7-positive cells (93). SiRNA-mediated silencing of *PAX7* in the latter led to increased expression of these myomiR. This suggests that PAX7 could downregulate myomiR expression levels in ARMS and contribute to its progression, which requires further study.

Both miR-1 and miR-206, sharing the same seed region sequence, were found to target *PAX3* in FN-RMS but not FP-RMS which is mostly likely due to the loss of the *PAX3* 3’UTR fragment upon fusion with *FOXO1* (31). Mir-1 and mir-206 were also found to target *Pax7* in mouse skeletal muscle satellite cells and the inhibition of these miRNA not only stabilizes *Pax7* expression but also inhibits myogenic differentiation and promotes cell proliferation (48). Pax3 and Pax7 transcription factors are known to be critical in activating the myogenic program whereby in their absence, muscle progenitor cells fail to enter myogenesis and complete differentiation into mature skeletal muscle fibers (94). However, their aberrant or sustained expression may prevent terminal differentiation of cells or contribute to tumorigenesis (48, 55, 95, 96). Moreover, using gene and proteomic profiling, low levels of PAX7 transcript and protein were found to be necessary for miR-206-mediated differentiation and cell cycle exit of FN-RMS cells only, which may be due to the absence of miR-206 binding sites in the fusion oncoprotein of FP-RMS cells (55). In addition to *PAX3/7*, key targets of miR-206 included *NOTCH3* and cyclin D2 (*CCND2*). However, since miR-206 is able to promote differentiation and cell cycle exit in both FN-RMS and FP-RMS subtypes, this indicates that miR-206 role in RMS tumorigenesis is not only acting through the regulation of PAX7 (55). Based on these studies, one can conclude that while miR-206 is capable of promoting RMS myogenic differentiation by different mechanisms of action, the upregulation of PAX7 in FN-RMS and the fusion of *PAX7* with *FOXO1* in FP-RMS are mechanisms by which RMS cells could evade miR-206-mediated differentiation and inhibition of cell cycle progression, therefore contributing to the aggressive behavior of this subtype. In turn, downregulating PAX7 expression levels promotes the expression of myomiR including miR-206 revealing an intricate interplay between genetic drivers of RMS tumorigenesis and myomiR function.

Significance of miR-1 and miR-206 in RMS has been attributed to the high levels of MET receptor, whose mRNA is a potential target of both miRNA in RMS tumors (49, 51). C-Met is a tyrosine kinase receptor and a transcriptional target of PAX3 (97). It is physiologically downregulated during myogenic differentiation but is overexpressed in both FN-RMS and FP-RMS, where its expression is upregulated by PAX3-FOXO1, and promotes metastatic behavior of tumor cells in both subtypes (97, 98). In fact, shRNA-mediated knockdown of *MET*, following induction with doxycycline, resulted in marked reduction of RMS xenograft tumor size in mice (97). Moreover, MET protein enhances RMS motility and metastatic propensity by activating downstream ERK signaling pathway (99). Its protein levels inversely correlated with miR-1 and miR-206 levels in RMS cells and the re-introduction of miR-1/206 into ERMS cells induced a downregulation of *c-Met* expression levels, which was found to have two target sites for miR-1/206 binding as revealed by bioinformatics analysis, as well as a significant reduction in the levels of phosphorylated ERK1/2 and FAK (49). miR-1/206 ectopic expression in RD cells (an ERMS cell line), in turn, decreased RMS proliferation and migration *in vitro* and tumor growth *in vivo* (49). Additionally, miR-206 re-expression in RMS induced myogenic differentiation and switched the neoplastic phenotype into one resembling skeletal muscle. This miRNA caused a reduction in Met levels to a level comparable to that of differentiating murine satellite cells, accompanied by an enhanced myogenic differentiation of both FP-RMS and FN-RMS cells (51).

Both miR-1 and miR-206 have binding sites in the 3’UTR region of *CCND2* and ectopic expression of either miRNA was able to downregulate CCND2 levels in RMS cells (31). CCND2 was shown to be a key regulator in promoting myogenic differentiation of muscle progenitor cells in dystrophin-deficient mice (100). RMS cells exhibit elevated expression of cell cycle regulator genes including *CCND2* compared to normal skeletal myocytes (101). While not demonstrated in this study, this suggests that miR-1/206-mediated silencing of *CCND2* could help reverse the incomplete myogenic differentiation of RMS cells (50).

MiR-1 has also been shown to inhibit *ZNF281*, a zinc finger transcription factor implicated in modulating cellular stemness, enhancing epithelial mesenchymal transition (EMT) in colon cancer cells (50, 102). ZNF281 can also promote differentiation of osteoblasts and neuronal cells but it inhibits the differentiation of muscle cells promoted by miR-1 (50, 103). In RMS patients, where miR-1 expression is low, ZNF281 transcript and protein expression levels are significantly high compared to their normal skeletal and smooth muscle tissue counterparts. It was therefore suggested that ZNF281 can be considered a marker of proliferative and dedifferentiation state of RMS and that its expression is partly regulated by miR-1 (50).

Generally, miR-206 seems to affect pathways that regulate RMS development, including the RAS/MAPK and NFκB signaling pathways as well as regulating myogenic transcription factors and epigenetic regulators (47). High miR-206 expression in FN-RMS was associated with a downregulation in interleukin-4 (*IL-4*) expression levels (52). IL-4 promoted RMS survival and proliferation while inhibiting MyoG expression (56).

MiR-206 targets also include *HDAC4*, a histone deacetylase enzyme that epigenetically inhibits gene expression by modulating the acetylation status of lysine residues on histone proteins thereby modifying chromatin structure (57, 104, 105). HDAC4 cellular localization has been shown to be important in regulating muscle differentiation (106). Oncomine database analysis shows its enrichment in RMS tissue compared to normal tissue and other sarcoma types (58, 107). It is mainly localized within the nucleus, in part due to Heme Oxygenase-1 (HO-1), which is an oxidative stress-response factor that promotes RMS progression by elevating downstream HGF and SDF-1 dependent pathways which stimulate myoblasts proliferation and inhibit their differentiation (58). RMS cells with elevated HO-1 produce less reactive oxygen species resulting in miR-206 repression. HO-1 inhibition, on the other hand, inhibits growth of RMS tumors by enhancing miR-206-mediated myogenic differentiation (58). HO-1 inhibition in RMS cells, promotes removal of HDAC4 from the nucleus, resulting in an upregulation of miR-206 and enhanced differentiation further revealing the tumor-suppressive effect of miR-206 in RMS (58).

### MiR-133

MiR-133 has been reported to impact myogenic differentiation but whether it promotes or inhibits differentiation is still a matter of debate and seems to be tissue-specific (83). To begin with, miR-133 was found to promote myoblast proliferation and acts conversely to miR-1 by inhibiting myocyte differentiation (108). In contrast, other studies showed that miR-133 members, miR-133a and miR-133b, inhibit myoblast proliferation while promoting myogenic differentiation in C2C12 cells by inhibiting ERK signaling (109, 110).

In both ERMS and ARMS, miR-133a levels, as those of miR-1, are reduced while their targets mRNA are upregulated (60). Furthermore, the reintroduction of either miRNA mimics into ERMS RD cells line appears to inhibit RMS growth. This was accompanied with a downregulation of several genes, all of which had isoforms that are highly expressed in striated skeletal and cardiac muscle. Additionally, the list of the top 50-downregulated targets for both miR-1 and miR-133a included the epigenetic regulators *HDAC4* and *SMARCD1* (60). The latter has been shown to regulate important players in muscle differentiation (111, 112). However, while ectopic expression of miR-1 promoted the expression of myogenic markers indicating a role in promoting myoblast differentiation, miR-133a did not (60). These effects observed in FN-ERMS were not as evident in FP-RMS cells whereby ectopic expression of miR-1/133a in Rh30 cells, a FP-RMS cell line, did not inhibit cell growth. The reason behind this has yet to be determined but may be due to the substantial levels of these myomiR in FP-RMS compared to FN-RMS or due to the presence of the fusion oncogene *PAX3-FOXO1* which may escape the miR-1/133a-mediated inhibition of cell growth.

Altogether, myo-miRs appear to be mostly tumor suppressive and act mainly by regulating myogenesis and promoting muscle cell differentiation. Their downregulation in RMS compared to normal skeletal muscle suggests their potential utilization in pro-differentiation therapy.

## Non myo-miRNA implicated in RMS

### MiR-28-3p and miR-193

In the context of SNAIL regulation of miRNA expression, analysis of the miRNA transcriptome in Rh30 cells revealed that *SNAIL* silencing can modulate miRNA levels (113). For example, *SNAIL* inhibition led to a downregulation of miR-28-3p and miR-193a-3p expression levels suggesting that SNAIL, which is upregulated in RMS tumors, leads to a downregulation of tumor-suppressive miRNA (113). Overexpression of these miRNA in Rh30 cells led to a potent inhibition of cell migration and a decrease in cell adhesion to endothelial cells (61). On another note, miR-193 family members act as tumor suppressors in many tumor types including colon cancer and osteosarcoma (114, 115). In another study, sixty myogenesis-related differentially expressed miRNA were identified in cells isolated from skeletal muscle biopsies, among which were miR-28-3p and miR-193a-5p that were shown to act as regulators of both ERMS and ARMS development and progression by downregulating proliferation, migration, invasion and contributing to cell cycle arrest (62). They were also able to induce the expression of myogenic transcription factors thereby promoting differentiation of RMS cells and myoblasts. Simultaneous overexpression of both miRNA diminished tumor growth in an *in vivo* model of RMS. Interestingly, miR-193a-5p overexpression in ARMS cells resulted in an upregulation of the myogenic-related miR-206 expression (62). Furthermore, miR-28-3p was found to indirectly downregulate EZR/ezrin in RMS, a key regulator of tumor metastasis (61, 113).

### MiR-29

MiR-29 family of miRNA, which includes miR-29a, miR-29b and miR-29c, while not specific to muscle tissue, is highly implicated in myogenesis (57, 63). They are all significantly downregulated in both FP-RMS and FN-RMS where they have been regarded as tumor-suppressive by promoting myogenic differentiation (31). MiR-29 expression levels are downregulated in RMS cells due to an upregulation in the NFκB-YY1 pathway which epigenetically silences miR-29 expression and blocks myoblast differentiation both *in vitro* and *in vivo* (63). NFκB enhances YY1 expression in RMS which then interacts with enhancer of Zeste homolog 2 (EZH2), a methyltransferase enzyme. This interaction downregulates mir-29 family members and inhibits myogenic differentiation (63). On the other hand, reconstitution of mir-29 exerts a negative feedback on YY1, inhibits RMS growth and induces myogenic differentiation in an RMS xenograft mouse model (63). Furthermore, inducing mir-29b expression resulted in elevated levels of differentiation markers in RMS cells and a phenotypic shift from a round to an elongated appearance (63). Additionally, overexpression of mir-29 using mimics in RMS cells inhibited proliferation, migration and invasion (64).

Moreover, in human RMS tumors, miR-29 expression levels were inversely correlated to those of cell cycle regulators *CCND2* and *E2F7* (31). Indeed, in transfected RMS cells, ectopic miR-29 expression did not only downregulate the aforementioned cell cycle regulators but also activated the expression of myogenic differentiation genes including MyoG and alpha-actin compared to non-transfected control. This was accompanied by an inhibition of proliferation of ERMS cells. In FP-ARMS cells, miR-29-mediated inhibition of proliferation was less prominent compared to FN-ERMS cells (31). Of note, while ectopic expression of all miR-29 members downregulated *CCND2* transcript levels, individual miR-29 family members exhibited cell-line specific regulation of CCDN2 protein levels. For example, miR-29a downregulated CCND2 protein levels in ERMS JR1 cells only. Similarly, all miR-29 family members were able to directly target 3’UTR of *E2F7* in HEK293 cells, but its levels were decreased upon ectopic expression of miR-29a in FN-ERMS JR1 cells and FP-ARMS Rh30 cells while there was no effect on *E2F7* expression in FN-ERMS RD cells. Similarly, for E2F7 protein levels, they were significantly downregulated in both JR1 and Rh30 cells by miR-29a, but no effect was observed in RD cells (31). Altogether, this indicated regulation of CCDN2 and E2F7 gene expression and protein levels by miR-29 family members in a cell-line specific manner. In the C2C12 mouse myogenic cell line, both mir-29 and mir-206 were demonstrated to not only inhibit *HDAC4* by directly targeting the 3’UTR of its transcript, but also inhibit the TGF-β-mediated upregulation of HDAC4 by targeting *Smad3*, a transducer of TGF-β signaling (57). Both TGF-β and Smad3 have been characterized as potent inhibitors of myogenic differentiation, and HDAC4 suppresses the activity of myogenic transcription factor MEF2 (57, 116–118). As such, these miRNA act against the inhibitory effect of TGF-β on myogenic differentiation. Furthermore, RMS cells express high levels of TGF-β and Smad4 associated with a downregulation in miR-29 and miR-206 levels which further supports the idea that these miRNA promote muscle cell differentiation by suppressing TGF-β signaling (57).

Another mechanism by which miR-29 exerts tumor suppressive effects in RMS is through targeting guanine nucleotide exchange factor T mRNA (*ARHGEF25/GEFT*), whose overexpression correlates with poorer outcomes (65). GEFT was shown to promote RMS cell survival and proliferation, migration, invasion and EMT thereby enhancing RMS metastatic propensity (64, 119). *GEFT* was verified as miR-29 target and its inhibition suppresses RMS proliferation and motility (64).

### MiR-450b-5p

Comprehensive microarray analysis on RMS cell lines and tissues identified a novel set of TGF-β1-related miRNA including miR-450b-5p as significantly expressed in TGF-β1 knocked-down RMS cells and TGF-β1-low-expression RMS tissues relative to control (66). In addition to its role in inhibiting myogenic differentiation, TGF-β1 is a known promoter of tumor cell metastasis and invasion in a variety of cancer types by modulating miRNA content (120). In RMS, TGF-β1 functions by suppressing miR-450b-5p, whereas miR-450b-5p significantly inhibits RMS growth and promotes the upregulation of MyoD *in vitro* and *in vivo* (66). Moreover, bioinformatics analysis revealed ecto-NOX disulfide-thiol exchanger 2 (*ENOX2*) and *PAX9* as candidate mRNA targets of miR-450-5p. Their expression inversely correlated with that of miR-450-5p in RMS cells, and their knockdown promoted MyoD expression as well. Altogether, TGF-β1 inhibits miR-450-5p in RMS which is associated with an upregulation in its downstream targets and repression of myogenic differentiation (66).

### MiR-221

MiR-221 is a tumor suppressive miRNA that is negatively regulated by PAX3-FOXO1 in FP-RMS (67). It functions partially through targeting *CCND2*, *CDK6*, and *ERBB3* mRNA. Its ectopic overexpression in FP-RMS induced a pro-apoptotic effect, reduced cell viability, migration and invasion in FP-RMS (67).

### MiR-7 and miR-324-5p

The overexpression of miR-7 and miR-324-5p inhibited cell proliferation and reduced tumor growth and metastasis in an *in vivo* RMS model (68). Additionally, miR-7 alone contributed to a decrease in cell invasion *in vitro*. Both miRNA have been described as tumor suppressive in several types of cancer and are regulators of alpha-9 integrin mRNA (*ITGA9*) in RMS (68, 121–124). Integrins mediate cell adhesion to the extracellular matrix thereby generating bidirectional signals mainly mediated by adapter proteins (125). ITGA9 has been shown to contribute to disease aggressiveness and metastasis in cancer (126). Furthermore, its inhibition is able to reduce motility and invasiveness in RMS cells (127).

### MiR-378-3p

MiR-378 family can target insulin-like growth factor receptor 1 (*IGF1R*) mRNA which is upregulated in both FN-RMS and FP-RMS and whose deregulated expression is associated with muscle diseases (69, 128). Ectopic overexpression of miR-378a-3p in Rh30 and RD cells suppressed IGF1R expression and downregulated IGF1R/AKT signaling (69). On a functional level, this was associated with a decrease in migration of RMS cells, an increase in apoptosis and cell cycle arrest and enhanced differentiation. Specifically, upregulation of miR-378a-3p in RMS cells using miRNA mimics led to a slight increase in muscle differentiation markers MyoD1 and MyHC, and a downregulation of myogenic-repressor MyoR and early-myogenic factor Myf5. More organized actin filaments were observed in miR-378a-3p-transfected cells resembling differentiated skeletal muscle. Altogether, miR-378-3p was able to modulate myogenic regulatory factors thereby promoting RMS differentiation. With that, treatment of RMS cells with 5-aza-2′-deoxycytidine was able to induce apoptosis, cell arrest at G2 phase and a decrease in migration associated with an upregulation in miR-378-3p levels (69).

### MiR-26a and miR-30b/30c

Comparing circulating serum levels of known tumor suppressive miRNA in RMS patients to healthy donors, miR-26a and miR-30b/30c were found lower in RMS patients (59). Furthermore, low levels of circulating miR-26a significantly correlated with progressive disease but this requires further study to determine whether circulating miR-26a could act as a biomarker in RMS patients (59).

### MiR-181a/212

A promyogenic miRNA cocktail (PMC) can contribute to myogenic epigenetic memory thereby influencing cell fate (70, 129). Treatment of murine FN-RMS with selected PMC increased expression of MyoG, a key transcription factor that promotes myogenic differentiation (130). *In silico* analysis identified miR-181a/212 as the key combination required for the observed pro-myogenic effect (70). *In vitro* studies showed that this combination increases the fusion index of MyHC-positive myotubes in FN-RMS cells while reducing their migration and proliferation capacity. Moreover, in a syngeneic murine transplant model of FN-RMS cell line pretreated with this selected miRNA cocktail in order to demonstrate whether treatment with PMC can promote myogenic commitment of FN-RMS and reduce its tumorigenicity, the mice injected with miRNA-pretreated cells showed a reduction in tumor size indicating a decreased proliferation of FN-RMS cells, and an improvement in functional activity of the mice (70).

### MiR-214

Evidence such as the downregulated levels of miR-214 in RMS cells and the inhibition of tumor growth mediated by its ectopic expression in RD cells, revealed that miR-214 is a tumor suppressive miRNA in RMS (71). Its downregulation is accompanied with an upregulation in N-Ras protein and transcript levels in murine embryonic fibroblasts, which acts as a proto-oncogene promoting tumor progression, and is demonstrated to be a target of miR-214. On the other hand, its ectopic expression was also able to induce differentiation and apoptosis while inhibiting colony formation and growth of xenografts *in vivo* (71).

### MiR-27a

MiR-27a is another tumor suppressive miRNA in RMS that can target *PAX3-FOXO1* by binding to *PAX3* mRNA as evidenced in mouse and human cells and can target both *PAX3* and *PAX7* mRNA in muscle cells (72). Indeed, pharmacological inhibition of HDAC3 decreased the activity of the chromatin remodeling enzyme SMARCA4 which is a chromatin remodeling enzyme. In turn, pharmacological or siRNA-mediated inhibition of *HDAC3* or *SMARCA4* promoted miR-27a expression. Re-expression of the latter decreased PAX3-FOXO1 in ARMS cells and reduced tumor growth, suggesting that miR-27a is tumor suppressive and part of the HDAC3-SMARCA4-miR27a axis (72).

While the above miRNA are tumor-suppressive whose dysregulated expression in RMS cells contributes to tumor growth and progression, miRNA discussed in the section below have been shown to promote RMS progression by regulating either tumor growth or motility or by correlating with advanced stage and poor prognosis in RMS patients, thereby acting as oncomiR.

## OncomiR implicated in RMS

Certain miRNA contribute to RMS metastasis, and can distinguish between FP-RMS and FN-RMS, raising their potential use as biomarkers of disease aggressiveness and possibly targets in RMS treatment strategies.

### MiR-27a

Analysis of the miRNA expression profile of FP-RMS and FN-RMS cell lines revealed 16 miRNA whose expression discriminates between FP and FN cell lines (73). One such miRNA is miR-27a which is upregulated in the more aggressive FP-RMS cell lines. miR-27a promotes proliferation and tumor progression in a variety of cancer types, suggesting an oncogenic role (131–136). Specifically, miR-27a was shown to promote tumor cell proliferation in RMS which was attributed to its role in targeting the retinoic acid receptors: retinoic acid alpha receptor (RARA) and retinoic X receptor alpha (RXRA) (73).

### MiR-183

miR-183 is significantly overexpressed in RMS where it can specifically target *EGR1* mRNA as validated *in vitro* (74). Its knockdown in different cancer cell lines, including RMS, leads to a deregulation of *miR-183–EGR1–PTEN* network by upregulating *EGR1* and *PTEN* mRNA levels, as well as EGR1 protein levels, both of which are tumor suppressive. While not studied in RMS particularly, miR-183 knockdown resulted in reduced cell migration through an *EGR1*-based mechanism. Therefore, miR-183 could be oncogenic in RMS, contributing to enhanced migration which is yet to be elucidated (74).

### MiR-9-5p

Another oncogenic miRNA in RMS is miR-9-5p, which has been associated with metastasis (75). In fact, miR-9-5p levels correlated with poor outcome, enhanced migration and metastasis in RMS patients. Particularly, its level of expression was modulated by the fusion oncoprotein PAX3-FOXO1 whereby reduction in the expression of the latter led to a decrease in miR-9-5p expression. This was attributed to PAX3-FOXO1-mediated regulation of its transcriptional target *MYCN* expression which in turn modulates that of miR-9-5p (75, 76).

### MiR-223

In both adolescent and young adult RMS patients, miR-223 was overexpressed and associated with an upregulation of both EMT and inflammation in an age-related manner, possibly contributing to RMS aggressiveness (77).

### MiR-17-92 cluster

Certain cases of ARMS, mostly FP-RMS, exhibit an amplification in a chromosomal region containing the *miR-17-92* cluster encoding several miRNA including miR-17, miR-19a/b, miR-20a, and miR-92 (78). While the majority of these cases were associated with an elevated expression of the miRNA, this upregulation was also found, to a lesser extent, in the absence of the chromosomal amplification. The upregulation of these miRNA correlated to poorer outcomes relative to non-amplified cases in neuroblastoma and its correlation with survival outcomes in RMS remains to be studied (137).

### MiR 130a/b

In a recent study, RNA-seq analysis for RMS of the head and neck revealed that *PPARG*, a critical gene in the PPAR-signaling pathway was downregulated and that miR-130a/b was highly expressed compared to normal tissue; this was further confirmed by western blot and qRT-PCR analysis, respectively (80). Bioinformatics analysis using the TARGET database revealed an interaction between miR-130a/b and *PPARG* and a negative correlation between their expression levels. This was confirmed *in vitro* in RD cells using antagomir to downregulate miR-130a/b levels which was associated with a significant reduction in RD cell proliferation. Treating RD cells with rosiglitazone maleate, a PPARG agonist, reduced proliferation and the combination of miR-130a/b antagomirs with rosiglitazone maleate significantly suppressed proliferation compared to RD cells treated with the agonist alone (80). Altogether, this indicates that miR-130a/b promotes RMS cell proliferation by targeting *PPARG* expression which requires further investigation.

### Exosomal miRNA

MiRNA enrichment within exosomes and their subsequent delivery into recipient cells suggests possible roles in modulating the tumor microenvironment to promote tumor growth. Exosomes are small extracellular vesicles which carry and deliver nucleic acid including non-coding RNA and protein cargos to recipient cells. They have been implicated in cancer progression in variety of tumor types where they essentially enhance recipient cell migration, invasion, anchorage-independent growth, and promote angiogenesis and immunomodulation (79, 138–142). miRNA enriched in ERMS-derived exosomes were represented in pathways implicated in cancer cell cycle biology, while those enriched in ARMS-derived exosomes targeted proteins implicated in cancer biology, metastasis, and stemness (138). Of note, while certain miRNA are enriched in exosomes consistent with their enrichment in the donor cells, others are only enriched in exosomes revealing selective sorting of these miRNA which may be independent of their level of expression in the donor cells (138, 143). Furthermore, 2 miRNA, miR-1246 and miR-1268, were commonly enriched in exosomes of all studied RMS cell lines. Bioinformatic pathway analysis revealed that they are related to tumorigenesis through regulating pathways including Wnt pathway, EGFR pathway, angiogenesis and apoptosis (138).

MiR-486-5p is implicated in several cancers where it either acts as a tumor suppressor or an oncomiR depending on tumor type and cellular context (67, 144–146). In RMS, miR-486-5p is enriched in both cells and exosomes, more particularly in FP-RMS (67). PAX3-FOXO1 upregulates the transcription of *miR-486-5p* which then promotes FP-RMS proliferation, colony formation and invasion. In fact, miR-486-5p inhibition reduced FP-RMS cell survival and decreased tumor growth in xenografted mice (67). Upregulating the expression of mir-486-5p in C2C12-derived exosomes, by transfecting C2C12 mouse myoblasts with either mir-486-5p or PAX3-FOXO1 expressing vectors, enhanced recipient fibroblast proliferation, migration, invasion and anchorage-independent growth (79). Additionally, analysis of a small series of human serum samples showed that miR-486-5p seems to be enriched in exosomes of RMS patients (79). However, specific effectors and downstream targets of miR-486-5p in RMS are yet to be determined.

### Other oncomiRNA

Utilization of the NanoString digital profiling technology in different prognostic groups of ERMS and spindle-cell sclerosing RMS (SRMS) patients revealed significant upregulation of either oncomiR or tumor suppressive miRNA (147). Of note, SRMS is biologically heterogeneous and like ERMS, it harbors a variety of genetic mutations that can result in different clinical outcomes. This study revealed differential expression of miRNA between the different prognostic groups. For example, miR-612, miR-3144-3p, miR-548y, miR-302d-3p, miR-421, miR-548y and miR-548ar-5p were significantly overexpressed in tumors with adverse/poor prognosis compared to those with favorable prognosis (147). These may be interesting miRNA players in RMS development and progression, but their specific roles in RMS have not been determined.

Therefore, oncomiR are important players in RMS progression and a better understanding of their roles and targets in RMS could pave the way for future treatment strategies that target these miRNAs or utilizes their expression levels for monitoring disease progression and treatment success.

## Long non-coding RNA implicated in RMS

**Figure 3:**
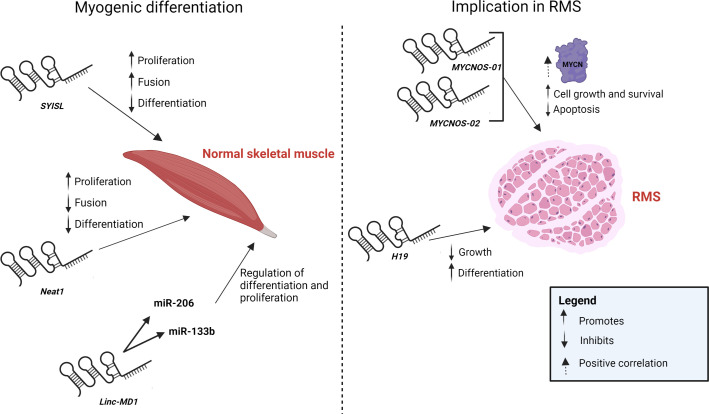
LncRNA implication in skeletal muscle myogenesis and RMS. Schematic diagram of lncRNA *SYISL*, *Neat1* and *Linc-MD1* implicated in skeletal muscle differentiation and proliferation (left panel) and lncRNA *MYCNOS-01*, *MYCNOS-02* and *H19* implicated in RMS proliferation biology (right panel). Created with BioRender.com.

LncRNA have been shown to play a role in regulating skeletal muscle differentiation. For instance, *SYISL* (SYNPO2-intron sense-overlapping), an abundant and intron-encoded lncRNA, was identified in muscle where it promotes myoblast proliferation and fusion (148) (**Figure 3**). However, it was demonstrated to inhibit myogenic differentiation. It acts by indirectly inhibiting MyoG, muscle creatine kinase (*MCK*), and myosin heavy chain 4 (*Myh4*) through the recruitment of EZH2 to their promoters. In fact, studies have shown that EZH2 interacts with various regulatory lncRNA and plays important roles in myogenesis (149). Similarly, lncRNA *Neat1* was shown to promote myogenic C2C12 division but inhibit their differentiation and fusion by recruiting Ezh2 to *p21* and to muscle-specific *Myog* and *Myh4* genes promoters (150) (**Figure 3**). Additionally, some lncRNA act as precursors of miRNA involved in myogenic differentiation. For example, the lncRNA *Linc-MD1* generates miR-206 and miR-133b indicating a role in muscle differentiation and a possible implication in RMS differentiation (41) (**Figure 3**). While these findings are general to skeletal muscle, determining the role of these lncRNA in RMS biology is worthy of examination.

In RMS, overexpression of the oncogene *MYCN* contributes to cell growth in ARMS where its transcription is driven by PAX3-FOXO1 (17, 76). The lncRNA *MYCNOS-01* and *MYCNOS-02* on the antisense strand of *MYCN* were shown to upregulate MYCN protein levels in RMS (**Figure 3**). However, *MYCNOS-01* did not affect mRNA levels and was presumed either to be acting as a cis-antisense lncRNA on its sense partner *MYCN*, or it may be interacting with a miRNA that targets *MYCN* thereby upregulating MYCN protein expression levels (17). In all cases, regulation of *MYCN via MYCNOS-01*, whose levels are upregulated in RMS cells harboring *MYCN* genomic amplification, was found to play a role in cell growth whereby silencing *MYCNOS-01* resulted in a reduction of MYCN protein levels in RMS cells, that in turn, specifically reduced cell viability of RMS cells having amplified *MYCN* expression (17). Similarly, a significant correlation exists between levels of *MYCNOS-02* and *MYCN* expression in the studied RMS samples and cell lines, however, knockdown of *MYCNOS-02* did not have consistent effects on *MYCN* expression. Decreasing *MYCNOS-02* expression, on the other hand, significantly downregulated cell survival but only in RMS cell lines with amplified *MYCN* expression, while promoting apoptosis. Altogether, the positive regulation of *MYCN* by *MYCNOS-01* and *MYCNOS-02* likely contributes to RMS cell growth (17).

*H19* lncRNA was among the first lncRNA whose function was described in various biological contexts. Expression of the *H19* gene is significantly suppressed in RMS tissue, and to a higher extent in ERMS, compared to normal muscle (151) (**Figure 3**). It is characterized by parental imprinting and a preferential loss of the maternal allele in RMS tissue and, as such, a loss-of-function which likely contributes to RMS development. *H19* has dual roles in cancer where it can promote tumorigenesis but can also act as a tumor suppressive lncRNA through different mechanisms of action (152–155). It is a precursor of miR-675, a miRNA embedded within its first exon, where its reactivation in RMS cells was shown to aid in the inhibition of RMS growth by upregulating *miR-675* expression and promoting muscle cell differentiation (156, 157).

## Circular RNA implicated in RMS

Some circRNA were shown to regulate myoblast differentiation. For instance, circLMO7 possesses the functional ability to act as a competing endogenous RNA for miR-378a-3p, which is involved in bovine muscle development; its overexpression stimulates proliferation and inhibits differentiation of primary bovine myoblasts (42). On the other hand, circFUT10 promotes myoblast differentiation but reduces their proliferation rate (158). This suggests that circRNA could be interesting players in myogenesis and in RMS differentiation which requires investigation.

**Figure 4:**
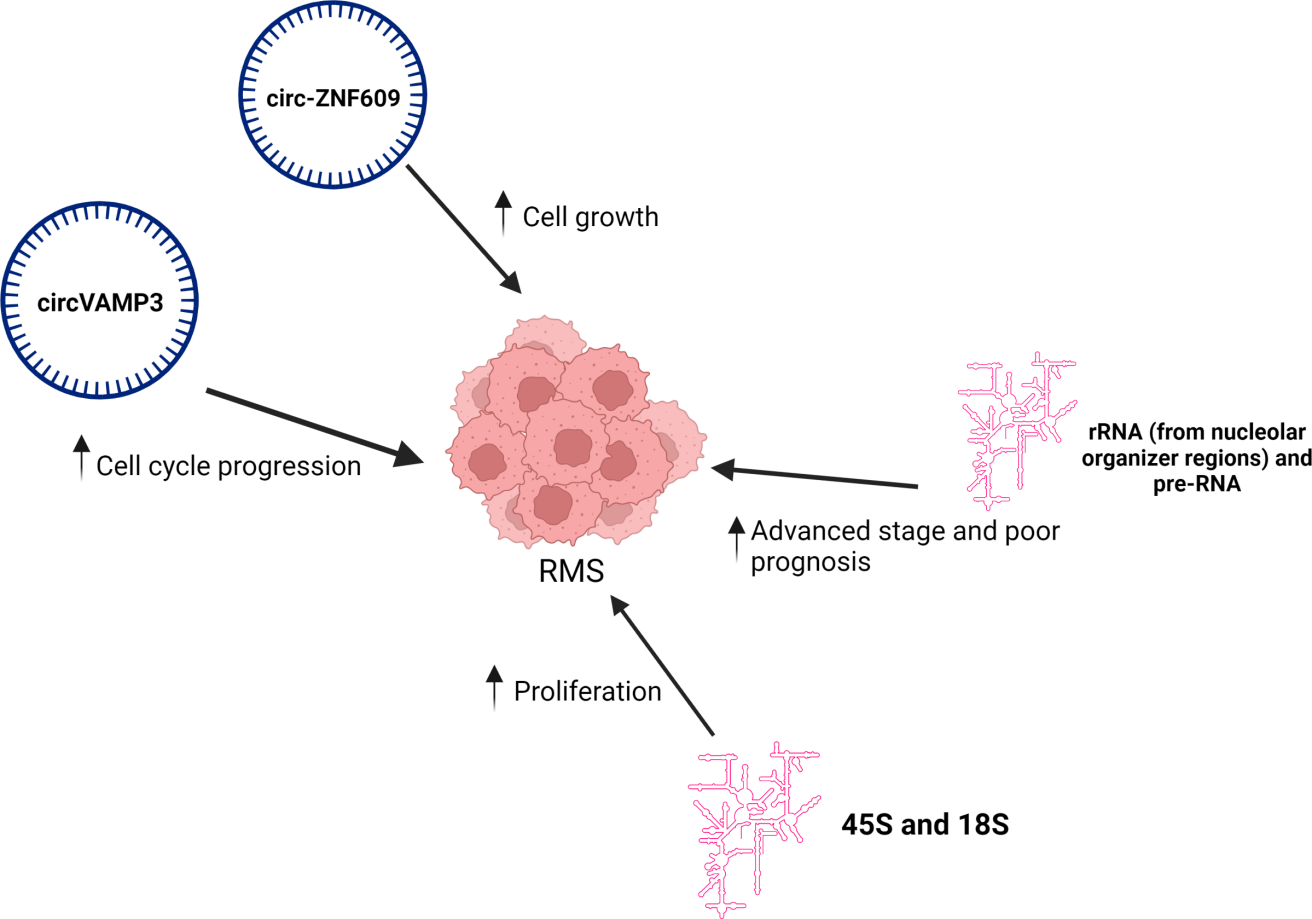
CircRNA and rRNA implicated in RMS biology. Schematic representation of circ-ZNF609 and circVAMP3 upregulating RMS cell growth and proliferation, respectively; rRNA from nucleolar organizer regions and pre-RNA correlated with advanced stage and poor prognosis of RMS; as well as ribosomal subunits 45S and 18S promoting RMS proliferation. Created with BioRender.com.

Two circRNA have been described to be implicated in RMS (**Figure 4**). Circ-ZNF609 has been shown to be upregulated in both ERMS and ARMS human biopsies (18). Its knockdown in the ERMS-derived cell line, but not in ARMS, induced cell cycle arrest at the G1/S transition, resulting in a strong reduction in p-Akt and pRb phosphorylation levels (18). This reveals a potential role for circ-ZNF609 in promoting RMS growth. Of note, high levels of circ-ZNF609 have been associated with tumor progression and poor prognosis in breast cancer (159). It was also shown to regulate myoblast proliferation (160). Its mouse homolog has been shown to inhibit myogenic differentiation by sponging miR-194-5p (161).

Another circRNA, circVAMP3, is significantly overexpressed in ARMS Rh4 cells compared to both normal myoblasts and the ERMS RD cell line (162). It was detected as both the circular and linear isoforms with the former being particularly more interesting to study in Rh4 cells because its upregulation was more prominent. Its depletion by siRNA technology, by targeting a unique site that distinguishes it from its linear counterpart, in the ARMS cell line impaired cell cycle progression leading to a small but significant increase in the percentage of cells in G2. *CircVAMP3* knockdown was shown to act through the alteration of the AKT-related pathway, and several AKT-pathway related genes were significantly deregulated such as AKT1 levels that were downregulated in siRNA-treated (si-circVAMP3) Rh4 cells compared to those treated with scrambled control. Furthermore, levels of the downstream inhibitors of the G2/M transition, the WEE1 and CDKN1A factors increased upon circVAMP3 knock-down (162).

## Ribosomal RNA implicated in RMS

An amplification of rRNA synthesis has been identified as a hallmark of cancer cell growth (163). While studies are still limited, certain rRNA have been implicated in RMS biology (**Figure 3**). A multifunctional protein and an indirect inhibitor of skeletal muscle differentiation, known as Prohibitin 2 (PHB2) was found to play an important role in ERMS RD cell line progression (19, 164). One of its methods of action was the regulation of rRNA; this was evidenced by the down-regulation of 45S and 18S rRNA synthesis following PHB2 knockdown. The exact mechanism behind this was a decrease in c-Myc occupancy at the ribosomal DNA (rDNA) promoter and an increase in MyoD molecules that were bound to it instead; altogether indicating that c-Myc-mediated rRNA synthesis is essential for ERMS cell proliferation (19). Moreover, comparative expressed sequence hybridization (CESH) suggested an increase in rRNA synthesized from the nucleolar organizer regions located on acrocentric chromosomes in RMS samples (165). This, in turn, correlated with poor prognosis, specifically in ARMS. In addition, pre-rRNA expression significantly correlated with tumor stage indicating that pre-rRNA could serve as a useful prognostic marker in ARMS.

## Therapeutic strategies and future implications

Extensive research has demonstrated the role of ncRNA in regulating myoblast differentiation and proliferation. In this review, we discussed how the dysregulated expression of ncRNA is associated with the incomplete myogenic differentiation that characterizes RMS cells as well as their enhanced proliferation and metastatic propensity. This indicates a potential use of ncRNA either in disease diagnosis and prognosis, as pro-differentiation agents, or as therapeutic targets in RMS treatment. In fact, to date, several ncRNA therapeutics in cancer and other diseases including the use of anti-miR and siRNA, have entered phase II or III of clinical development, though not yet in RMS (166). For example, Miravirsen is an anti-miR-122 that has entered phase II clinical trials against Hepatitis C viral infection and the antisense oligonucleotide Prexigebersen has also entered phase II clinical trials in acute and chronic myeloid leukemia patients (166).

Clinical trials investigating miRNA as biomarkers include those that monitor miRNA levels in patients receiving FDA-approved drug treatment. The advantage of using miRNA as biomarkers is their ubiquitous expression in different body fluids including serum and saliva (167). Differentially expressed miRNA between FP-RMS and FN-RMS may serve as biomarkers that distinguish between the two subtypes, as well as for prognostication. For example, low miR-206 expression correlated with poor overall survival in metastatic RMS cases lacking the PAX3-FOXO1 fusion protein (52). Their circulation and detection in body fluids is especially important in allowing their use in monitoring disease progression and treatment success. We mention, for example, how comparing serum levels of circulating miR-26a and miR-30b/30c distinguishes between RMS patients and healthy donors as these miRNA are detected at lower levels in RMS patients (59). This indicates that these miRNA have a potential role in patient diagnosis. In fact, studies suggest that ncRNA, particularly the expression signature of several ncRNA seems to be promising in cancer diagnosis and prognosis (168).

Pro-differentiation therapy has been clinically established in cancer and the role of myomiR in promoting skeletal myoblast differentiation suggests that they may be essential players in this kind of therapy (60, 169). Ectopic expression of cell-type specific miRNA can shift the mRNA expression profile to one resembling the tissue or cell types in which the miRNA are originally enriched (51, 170). For example, pre-miR-1 and pre-miR-206 induced myogenic differentiation and switched the mRNA expression profile in ERMS (RD18) and ARMS (RH4) mice xenografts into one resembling mature skeletal muscle cell (51). One way to enhance the expression of a certain miRNA is through the use of miRNA mimics, synthetic versions of endogenous miRNA that can restore the regulation of gene expression (171). Hanna *et al.*, demonstrated that the knockdown of a single or a subset of miR-206 targets was insufficient in promoting differentiation of RMS cells compared to the treatment with miR-206 mimics which can both promote differentiation and cell cycle exit suggesting that because a miRNA can have hundreds of targets, miRNA replacement therapy may be more valuable than therapies that target single molecules or pathways (55). Interestingly, expression of miRNA may be dysregulated in cancer due to aberrant epigenetic events including global hypermethylation observed in cancer cells (172). With that, studies have shown that hypomethylating agents, in addition to histone deacytelase inhibitors, can activate miRNA expression in different types of cancer. For example, treatment of bladder cancer cells with the prodrug 5-aza-2- deoxycytidine (Decitabine), a methyltransferase inhibitor/hypomethylating agent, and 4-phenylbutyrate induced high expression of miR-127 with tumor suppressive role in bladder cancer cells (172).

Small molecules comprise another approach to regulate ncRNA. The advantages of small molecules in RNA therapeutics are their low molecular weights, oral administration, and permeability that allows them to easily pass the cell membrane (173). Their synthesis is also more cost-effective compared to synthetic oligos. They can act by interacting with proteins involved in ncRNA synthesis thereby regulating ncRNA expression (173). For example, enoxacin is a small molecule identified to enhance miR-125a expression by acting at the level of pre- and pri-miRNA (174). However, in addition to problems with delivery and efficacy, the fact that miRNA influence the expression of thousands of genes may also pose a problem due to possible off-target effects. Therefore, the use of miRNA to promote myogenic differentiation requires extensive understanding of their mechanisms of action in regulating myogenesis and ensuring that their ectopic expression does not exceed physiological levels (169, 175).

Targeting ncRNA expression, on the other hand, involves RNA interference (RNAi) therapy which includes the inhibition of oncomiR by utilizing synthetic anti-miR also known as blockmir or antagomir. These bind to complimentary miRNA and inhibit their activity or mask the binding site of certain miRNA targets, respectively (171). Therefore, targeting oncomiR in RMS may help to reduce proliferation, migration and/or invasion. Small molecule inhibitors can also be used to target miRNA expression. For example, Targaprimir-96 is a miRNA-processing inhibitor which can bind to pri-miR-96 and inhibit its processing into mature oncogenic miR-96 (176). However, the best miRNA targets for RMS treatment have to be identified and one method is through collection of biopsy samples from a large number of patients and at different stages of disease progression to determine key miRNA and ideal targets for treatment (177). CircRNA therapeutics also includes regulating circRNA expression through their targeting, for example by the use of gold nanoparticles conjugated with siRNA, or through overexpression of tumor-suppressive circRNA (178). This has shown to downregulate cancer progression and enhance response to chemotherapeutic treatment. LncRNA targeting includes inhibition of lncRNA transcription, modulation of genomic loci that encode lncRNA or post-transcriptional inhibition (166). These strategies have yet to be explored in RMS biology.

Altogether, correction of dysregulated ncRNA could be a promising intervention that can be adjuvant to existing standard treatment strategies against RMS which is needed to enhance survival rates, but the efficient delivery and ensuring safety of the drugs that target ncRNA should be improved and studies are needed to establish their efficacy in RMS.

## Conclusion

miRNA and other ncRNA are important players in RMS tumorigenesis. The specific role of miRNA in muscle growth and differentiation whereby dysregulation, even in small amounts, in their expression can determine muscle differentiation reveals important mechanistic roles of these miRNA in RMS biology. We have summarized the evidence to date showing that the dysregulation of miRNA is crucial in RMS and how these miRNA may serve in important prognostic roles and in ncRNA-based therapeutic strategies in RMS patients. However, sufficient studies that include large patient cohorts, RMS serum specimens, and clinical samples revealing the diagnostic and prognostic potential of miRNA in RMS are still lacking. Furthermore, lncRNA, circRNA and rRNA are emerging key players in cancer growth and progression, but these are largely understudied in RMS biology thereby necessitating further investigation.

## Author contributions

FR, RS and SG drafted and refined the manuscript. NH, PC and PAC reviewed and critically read the manuscript. All authors contributed and approved the submitted version.

## Funding

This work was funded by a grant from the Lebanese University, a grant from Ligue Contre le Cancer (Rhone) and a grant from Aix-Marseille University.

## Conflict of interest

The authors declare that the research was conducted in the absence of any commercial or financial relationships that could be construed as a potential conflict of interest.

## Publisher’s note

All claims expressed in this article are solely those of the authors and do not necessarily represent those of their affiliated organizations, or those of the publisher, the editors and the reviewers. Any product that may be evaluated in this article, or claim that may be made by its manufacturer, is not guaranteed or endorsed by the publisher.
